# An Antidepressant Drug Increased TRAIL Receptor-2 Expression and Sensitized Lung Cancer Cells to TRAIL-induced Apoptosis

**DOI:** 10.2174/0118715206262252231004110310

**Published:** 2023-12-04

**Authors:** Kazi Mohammad Ali Zinnah, Ali Newaz Munna, Jae-Won Seol, Byung-Yong Park, Sang-Youel Park

**Affiliations:** 1 Biosafety Research Institute, College of Veterinary Medicine, Jeonbuk National University, Gobong ro, Iksan, Jeonbuk, 54596, South Korea;; 2 Department of Animal and Fish Biotechnology, Faculty of Biotechnology and Genetic Engineering, Sylhet Agricultural University, Sylhet, 3100, Bangladesh

**Keywords:** Desipramine, autophagy, apoptosis, death receptor-5, TRAIL, antidepressant drug

## Abstract

**Background:**

TRAIL has emerged as a promising therapeutic target due to its ability to selectively induce apoptosis in cancer cells while sparing normal cells. Autophagy, a highly regulated cellular recycling mechanism, is known to play a cell survival role by providing a required environment for the cell. Recent studies suggest that autophagy plays a significant role in increasing TRAIL resistance in certain cancer cells. Thus, regulating autophagy in TRAIL-mediated cancer therapy is crucial for its role in cancer treatment.

**Objective:**

Our study explored whether the antidepressant drug desipramine could enhance the ability of TRAIL to kill cancer cells by inhibiting autophagy.

**Methods:**

The effect of desipramine on TRAIL sensitivity was examined in various lung cancer cell lines. Cell viability was measured by morphological analysis, trypan blue exclusion, and crystal violet staining. Flow cytometry analysis was carried out to measure apoptosis with annexin V-PI stained cells. Western blotting, rtPCR, and immunocytochemistry were carried out to measure autophagy and death receptor expression. TEM was carried out to detect autophagy inhibition.

**Results:**

Desipramine treatment increased the TRAIL sensitivity in all lung cancer cell lines. Mechanistically, desipramine treatment induced death receptor expression to increase TRAIL sensitivity. This effect was confirmed when the genetic blockade of DR5 reduced the effect of desipramine in enhanced TRAIL-mediated cell death. Further investigation revealed that desipramine treatment increased the LC3 and p62 levels, indicating the inhibition of lysosomal degradation of autophagy. Notably, TRAIL, in combination with either desipramine or the autophagy inhibitor chloroquine, exhibited enhanced cytotoxicity compared to TRAIL treatment alone.

**Conclusion:**

Our findings revealed the potential of desipramine to induce TRAIL-mediated cell death by autophagy impairment. This discovery suggests its therapeutic potential for inducing TRAIL-mediated cell death by increasing the expression of death receptors, which is caused by impairing autophagy.

## 1 INTRODUCTION

Lung cancer is one of the most prevalent and deadliest forms of cancer, causing a substantial number of deaths worldwide [1, 2]. Among the cancers detected in the USA in 2018, lung cancer ranked second in terms of incidence rate [3]. Approximately 1.6 million lives are lost due to lung cancer annually. Moreover, around 1.8 million people are newly diagnosed each year [4]. After diagnosis, the 5-year survival rate is 4-17%, which varies based on the stage of the cancer [5]. Various treatment strategies include targeted chemotherapeutic agents, surgery, and radiotherapy to treat advanced non-small cell lung cancer [6, 7]. Newer approaches, such as chemotherapeutic drugs in targeted adjuvant approaches, hold promise as a viable option for cancer treatment, including lung cancer [8, 9].

Tumor necrosis factor-related apoptosis-inducing ligand (TRAIL) is a transmembrane cytokine that has shown the prospect of being used successfully for cancer treatment [10]. It is able to target a wide range of tumor cells without affecting normal cells [11]. TRAIL-mediated cancer cell killing can occur *via* both extrinsic and intrinsic apoptotic pathways [12]. TRAIL triggers apoptotic signals through bindings to its death receptors, (DR4 and DR5) [12, 13]. The interaction between TRAIL and its receptors triggers the recruitment of the Fas-associated death domain (FADD), facilitating the subsequent recruitment of procaspase-8 [14]. This activates the death-inducing signaling complex (DISC), triggering the activation of caspases-8 and -9, leading to the activation of the effector caspases-3, -6, and -7. Consequently, various cellular changes occur, including membrane blebbing, DNA fragmentation, and nuclear shrinkage [15, 16]. Although TRAIL is unique for its cancer cell-killing capacity, various cancer cells are resistant to TRAIL [17, 18]. The apoptotic effect of the TRAIL is ineffective against numerous tumor cells, including human A549 lung cancer cells, due to their resistance [19, 20]. However, effective TRAIL-sensitizing agents have been demonstrated in several studies to have the potential to overcome TRAIL resistance [21].

Autophagy, also known as programmed cell death type II, is a key cellular mechanism to maintain cellular homeostasis and has been posited as an alternative cell death mechanism [22, 23]. Autophagy eliminates cytosolic components and damaged or misfolded proteins using a lysosome-mediated degradation system, which is promoted under stress conditions, such as starvation, hypoxia, growth factor deprivation, and endoplasmic reticulum stress [24, 25]. Autophagic flux is the complete mechanism of autophagy that involves the sequestration of cytosolic components into a double-membrane vesicle called an autophagosome, followed by fusion with the lysosome [26]. The acidic pH and lysosomal enzymes initiate the degradation and recycling of these cytosolic components [27]. During the formation of autophagosome, the microtubule-associated protein, light chain 3 (LC3)-I, is converted into its lipid-conjugated form, LC3-II [28]. This conversion is commonly considered a marker of complete autophagosome formation [29, 30]. The autophagosome then combines with lysosomes. p62 (SQSTM1), a ubiquitin-binding protein, is localized in autophagosomes and commonly used together with LC3- II to identify autophagy induction or inhibition. Thus, an elevated p62 level marks an inhibition of autophagy [31, 32]. Autophagy initially acts as a tumor suppressor during tumor formation in healthy conditions, but once the tumor forms, it can facilitate cancer cells, making autophagy a double-edged sword [23, 33]. Numerous studies have described the protective mechanism of autophagy by providing necessary energy during metabolic stress and preventing cancer cell death [34, 35]. Recent findings have revealed that inhibition of autophagy, either through pharmacological means or genetic manipulation, enhanced cancer cell death during chemotherapy, indicating that autophagic flux inhibition might be a suitable and promising strategy for cancer treatment [36-38]. For example, chloroquine (CQ), or related hydroxychloroquine (HCQ), is an autophagy inhibitor that prevents the acidification of lysosomes, inhibits the fusion of autophagosomes with lysosomes, and augments the apoptotic effect [39-41].

Antidepressants are commonly recommended for the treatment of depression, psychiatric disorders, and chronic pain in cancer patients [42]. For example, desipramine, a tricyclic antidepressant (TCA), is used as a first-line drug to treat neuropathic pain [43]. As a member of the TCA class of drugs, desipramine has shown cytotoxic effects in many cancer cell lines, such as human MG63 osteosarcoma cells [44], human HT29 colon carcinoma cells [45], human PC3 prostate cancer cells [46], C6 glioma cells [47], and mouse Ca3/7 skin squamous cells [48]. However, to date, there has been no report on the anti-cancer effect of desipramine, specifically on lung cancer cells. Therefore, we explored the potential therapeutic effect of desipramine in lung cancer cells.

Our present study demonstrated the role of autophagy flux inhibition by desipramine, which enhanced TRAIL-mediated apoptosis in lung cancer cells due to elevated expression of DR5. Notably, individual treatment with either desipramine or TRAIL did not affect cell viability.

## 2 MATERIALS AND METHODS

### 2.1 Cells and Culture Systems

A549 and HCC-15 lung cancer cell lines were kindly provided by the American Type Culture Collection (Global Bioresource Center, Manassas, VA, USA). The Calu-3 cancer cell line was purchased from the Korean Cell Line Bank (Korean Cell Line Research Foundation). All cell lines were cultured in Roswell Park Memorial Institute-1640 medium (Gibco BRL, Grand Island, NY, USA), supplemented with 10% (v/v) fetal bovine serum and antibiotics (100 μg/mL penicillin-streptomycin) at 37°C in a 5% CO_2_ incubator.

### 2.2 Reagents

Desipramine and CQ were purchased from Sigma-Aldrich (St. Louis, MO, USA), and TRAIL (100 ng/mL) was purchased from AbFrontier (Geumcheon-gu, Seoul, the Republic of Korea).

### 2.3 Cell Viability Assay

MTT and crystal violet staining were conducted for cell viability measurement. Cells were given pretreatment with desipramine (different doses) and/or CQ (20 μM) for 12 h and then exposed to recombinant TRAIL (100 ng/mL) for an additional 3 h. Cell morphology was captured under an inverted microscope (Nikon, Tokyo, Japan), and MTT and Crystal violet staining assays were done following the protocol as previously described [49].

### 2.4 Trypan Blue Exclusion Assay

Viable cells were counted using microscopy and a hemocytometer after staining the cells with trypan blue (Sigma-Aldrich). The results were calculated as percentages and compared to those of the vehicle-treated controls. Protocol was carried out as previously described [50].

### 2.5 Colony-formation Assay

A549 lung cancer cells were cultured in 12-well plates and treated with different doses of TRAIL and desipramine. The procedure was done as previously described [51].

### 2.6 Apoptosis Measurement Assay

Apoptosis was assessed by flow cytometry using an Annexin V Assay Kit (Santa Cruz Biotechnology, Santa Cruz, CA, USA), according to the manufacturer's protocol. Annexin V levels were determined by measuring fluorescence at 488 nm of excitation and 525/30 emission using a Guava easyCyteHT System (Millipore, Bedford, MA, USA) as previously described [51].

### 2.7 Western Blot Assay

Western blot analysis was carried out following the previously described protocol [52]. Cells were lysed in a lysis buffer, followed by centrifugation at 11,200×g to collect the protein samples by removing the pellet. The proteins were then separated in an SDS-PAGE gel and transferred onto a nitrocellulose or PVDF membrane. After blocking, the membrane was incubated with the primary antibody for one hour at room temperature. Primary antibodies, such as DR5 (1:10,000; Abcam, Cambridge, MA, USA), DR4 (1: 1,000; Abcam), LC3 (1: 1,000; Sigma-Aldrich), p62 (Sigma-Aldrich), atg5 (Cell Signaling Technology, Danvers, MA, USA), cleaved caspase-3 (Cell Signaling Technology), cleaved caspase-8 (BD Pharmingen/BD Biosciences, San Jose, CA, USA), and β-actin (Sigma-Aldrich), were detected. Followed by a secondary antibody probing, the bands were visualized using a Fusion-FX7 imaging system (Vilber Lourmat, Marne-la-Vallée, France).

### 2.8 Immunocytochemistry (ICC)

ICC was carried out following the previously described protocol [53]. Cells grown on glass coverslips were treated with desipramine, fixed with paraformaldehyde, and permeabilized with Triton X-100. After blocking with BSA, cells were incubated with primary antibodies against p62 and DR4/5, followed by incubation with secondary antibodies. Finally, cells were stained with DAPI, mounted on slides, and observed under a fluorescence microscope (Nikon ECLIPSE 80i) at 400x magnification.

### 2.9 Transmission Electron Microscopy (TEM)

Samples were prepared as described before [54]. Thin sections with a thickness of 60 nm were prepared using an LKB III ultramicrotome from Leica Microsystems GmbH (Wetzlar, Germany). These sections were stained with 0.5% uranyl acetate (Electron Microscopy Sciences) for 20 minutes and 0.1% lead citrate (Electron Microscopy Sciences) for 7 minutes at room temperature. Subsequently, the sections were examined under a Hitachi H7650 electron microscope (Hitachi, Ltd., Tokyo, Japan) with a magnification of ×10,000, located at the Center for University-Wide Research Facilities at Jeonbuk National University.

### 2.10 Small Interfering RNA (siRNA) Transfection

Tested cell lines were transfected with siRNA using Lipofectamine 2000 (Invitrogen) according to the manufacturer's protocol. Knockdown efficiency was assessed by immunoblotting. DR5-specific and scrambled control siRNA were purchased from Ambion, Life Technologies; atg5-specific siRNA and transfection reagent Lipofectamine 2000 were purchased from Invitrogen. This was done by the method used in the previous study [55].

### 2.11 Quantitative Reverse Transcription Polymerase Chain Reaction (qRT-PCR)

Protocol was carried out following the method described in the previous study [52]. Total RNA was extracted using RiboEX (GeneAll Biotechnology, Korea) buffer. The extracts were then converted into cDNA using reverse transcriptase (Enzynomics, Korea) on a CFX96^TM^ Real-PCR Detection System (Bio-Rad Laboratories), following the manufacturer’s instructions. Gene primers (1 µL), with SYBR Green (Bio-Rad Laboratories) and a total reaction volume of 20 µL, were used for qRT-PCR. The sequences of the primers used were DR5 (forward: 5'-GCGGTCCTGCTGTTGGTCTC-3', reverse: 5'-GCTTCTGTCCACACGCTCAG-3') and GAPDH, which was used as an internal control (forward: 5'-TGCACCACCAA CTGCTTAG-3', reverse: 5'-GGATGCAGGGATGATGTT-3'). All data were evaluated using Bio-Rad CFX manager version 2.1 analysis software (Bio-Rad Laboratories).

### 2.12 Statistical Analysis

Data are presented as mean ± standard deviation (SD). Statistical analysis was performed using one-way analysis of variance (ANOVA), followed by the Tukey-Kramer test. GraphPad Prism 5 software (GraphPad Software, Inc.) was used for statistical analyses. A *p*-value of less than 0.05 was considered statistically significant.

## 3 RESULTS

### 3.1 Effects of Desipramine Treatment on TRAIL-induced Death of Lung Cancer Cells

**Fig. (1):**
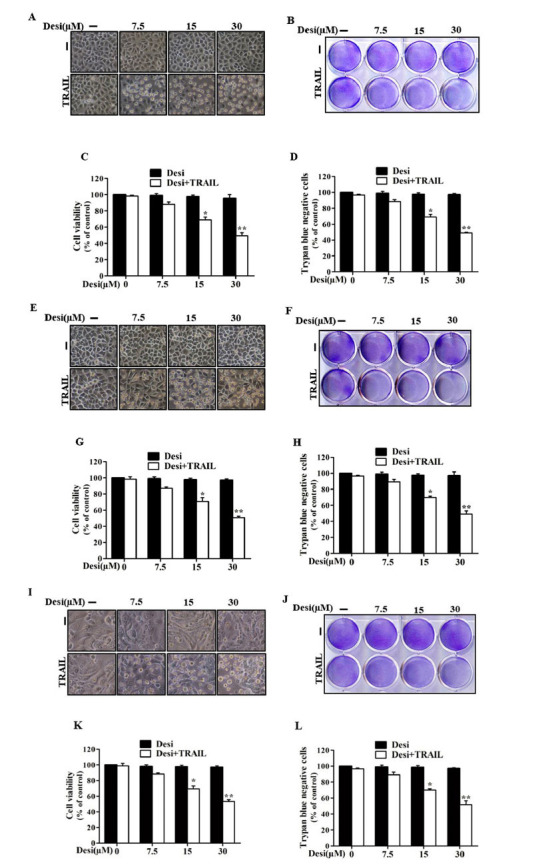
Effects of desipramine treatment on TRAIL-induced apoptosis of lung cancer cells. (**A**-**D**) A549, (**E**-**H**) HCC-15, and (**I**-**L**) Calu-3 cells were preincubated with designated concentrations of desipramine for 12 h and then with TRAIL (100 ng/mL) for 3 h. (**A**, **E**, and **I**) Cells were photographed, and variations in morphology were examined under a light microscope (×100). (**B**, **F**, and **J**) The Mean density of crystal violet stained was analyzed. (**C**, **G** and **K**) MTT assays were conducted to analyze cell viability, as represented by a bar graph. (**D**, **H** and **L**) Viable cells were counted by trypan blue exclusion assay. Statistically significant differences between the control and each indicated treatment group are shown as *p <0.01 and **p <0.001. Each experiment was repeated three times. Desi: desipramine; TRAIL: tumor necrosis factor-related apoptosis-inducing ligand.

We aimed to investigate the synergistic effect of desipramine on TRAIL sensitivity in lung cancer cells. Our experimental results indicated a robust combined effect of desipramine in all three lung cancer cell lines tested (A549, HCC-15, and Calu-3). In this experiment, we treated the cells with desipramine (30 µM) for 12 h and then co-treated with TRAIL (100 ng/mL) for a further 3 h. After that, we captured the morphological changes using a light microscope (Fig. **1**). We observed significant TRAIL-mediated cytotoxicity in desipramine-treated cells (Figs.**1A**, **E** and **I**), and the density of crystal violet dye decreased due to apoptotic cell death (Figs. **1B**, **F** and **J**). This was further determined by MTT assay showing substantial inhibition of cell growth in the co-treated group in a dose-dependent manner (Figs.**1C**, **G** and **K**). The trypan blue exclusion assay showed that the combined treatment, compared to a single treatment, robustly decreased the number of viable cells to a greater extent (Figs. **1D**, **H** and **L**). These findings indicated that desipramine induced TRAIL sensitivity in the TRAIL-resistant lung adenocarcinoma cells to TRAIL-mediated apoptotic cell death.

### 3.2 Combined Desipramine and TRAIL Treatment Effectively Inhibited the Formation of A549 Cell Colonies and Enhanced TRAIL-mediated Apoptosis

**Fig. (2):**
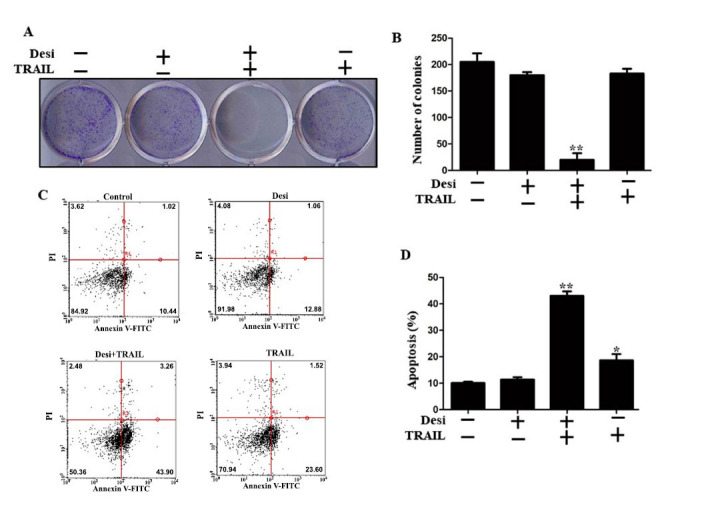
Combined desipramine and TRAIL inhibit colony formation and enhance TRAIL-mediated apoptosis. (**A**, **B**) A549 cells were cultured with the indicated doses of desipramine (30 μM) and/or TRAIL (100 ng/mL). After 24 h, the medium was replaced with new medium without drugs and further cultured for a week. Colonies were stained with crystal violet dye and counted. (**C**, **D**) Cells were pretreated with desipramine (30 μM) for 12 h and then treated with TRAIL (100 ng/mL) for an additional 2 h 30 min. Apoptosis was assessed by annexin V assay after using 1:1 fluorescein isothiocyanate and propidium iodide reagents. Statistically significant differences between the control and each indicated treatment group are shown as *p <0.05 and **p <0.001. The results represent the mean of three independent experiments.

We further investigated the combination effects of desipramine and TRAIL on the colony-forming capacity of A549 cancer cells. When A549 cells were cultured with desipramine (30 µM) for 3 days, colony formation was completely inhibited; thus, the desipramine dose was reduced to 15 µM. Single TRAIL or desipramine treatment slightly reduced colony formation, whereas combined TRAIL-drug treatment significantly reduced colony formation and size (Figs. **2A**, **B**). Annexin V-PI analysis proved that desipramine and TRAIL co-treatment resulted in significantly higher augmentation of apoptotic cell death compared to treatment with either desipramine or TRAIL individually (Figs. **2C**, **D**). These results confirmed that desipramine increased the TRAIL-induced apoptosis in the TRAIL-resistant A549 cells.

### 3.3 Effects of TRAIL Receptor-2 (DR5) on TRAIL-induced Apoptosis

**Fig. (3):**
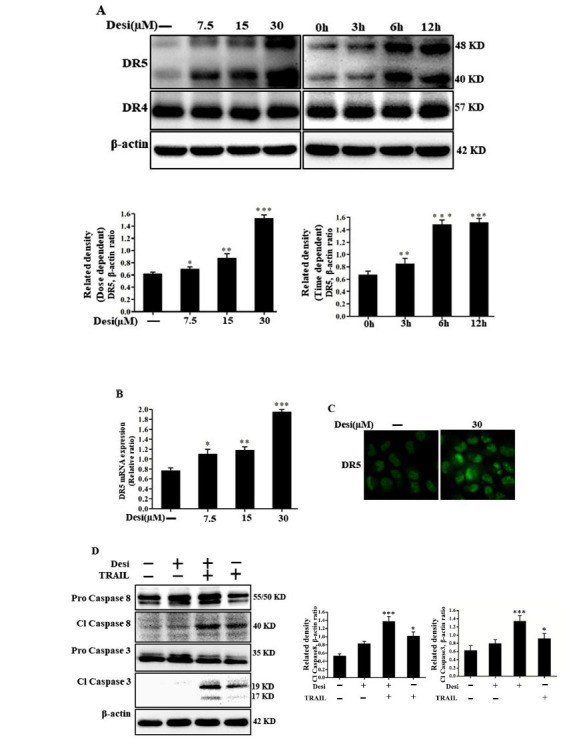
Effects of TRAIL receptor-2 (DR5) on TRAIL-induced apoptosis. A549 cells were preincu bated with designated doses of desipramine for 12 h. (**A**) Harvested cell lysates were collected and subjected to western blot analysis to measure DR4 and DR5 expression levels. (**B**) DR5 mRNA expression was assessed by qRT-PCR. (**C**) ICC revealed substantial DR5 expression in desipramine-treated cells. (**D**) Desipramine (30 μM)-treated cells were incubated for 12 h and then exposed to TRAIL (100 ng/mL) for 3 h. The intracellular apoptosis regulatory proteins, cleaved caspase-8 and cleaved caspase-3 were detected by immunoblot analysis. Every experiment was repeated at least twice. Statistically significant differences between the control and each indicated treatment group are shown as*p <0.05, **p <0.01 and ***p <0.001.

To understand the molecular basis of increased TRAIL sensitivity in A549 cells by desipramine, we considered whether the expression of death receptors expression was involved in TRAIL sensitivity. TRAIL resistance in several cancer cells was due to the decreased expression of the TRAIL receptors DR4 and DR5 (containing death domain) or increased decoy receptors DcR1 and DcR2 expression [56, 57]. Western blot analysis of the whole cell lysates revealed that desipramine treatment enhanced DR5 expression both dose and time-dependently, although there was no significant change in DR4 expression (Fig. **3A**). Desipramine treatment also increased DR5 transcript levels (Fig. **3B**). Furthermore, immunocytochemistry (ICC) results revealed a substantially greater appearance of DR5 in cells treated with desipramine compared to untreated cells (Fig. **3C**). Finally, the induced apoptosis was confirmed by elevated cleaved caspase-8 and cleaved caspase-3 levels in the combination group compared to the only TRAIL-treated group (Fig. **3D**). Therefore, DR5 potentiation by desipramine is essential for TRAIL-induced apoptosis.

### 3.4 Suppression of DR5 Altered the Results of Desipramine-induced TRAIL-mediated Apoptosis

**Fig. (4):**
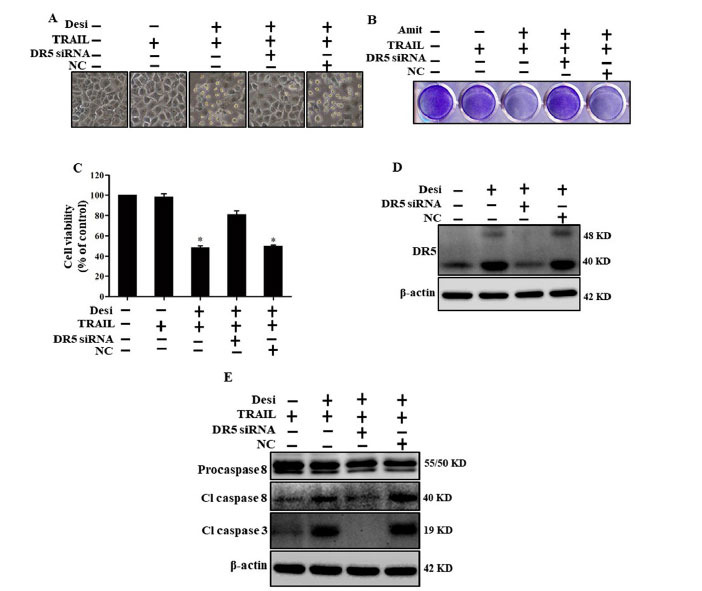
Suppression of DR5 reverses desipramine-induced TRAIL-mediated apoptosis. DR5-specific siRNA and NC siRNA (40 nM) were transfected into cells for 24 h. The cells were then treated with desipramine (30 μM) for 12 h and finally exposed to TRAIL (100 ng/mL) for 3 h. (**A**) Cells were photographed, and morphological variations were examined under a light microscope (×100). (**B**) Mean density of crystal violet stained A549 cells is shown. (**C**) MTT assays were conducted to calculate cell viability percentages, as represented by a bar diagram. Statistically significant differences between the control and each indicated treatment group are shown as *p <0.001. (**D**) Cell lysates were harvested and subjected to western blot analysis to determine DR5 expression. (**E**) Apoptosis indicator protein signals were evaluated by western blot analysis. β-actin was used as a loading control. Each experiment was repeated three times for (Figs. **A**-**C**) and twice for (Figs. **D**, **E**).

We applied DR5-specific siRNA to block DR5 expression, thereby restoring cancer cell viability. We discovered the pivotal role of DR5 in enhancing the effect of desipramine on TRAIL-induced apoptosis. After transfection with DR5-specific siRNA or negative control (NC) siRNA for 24 hours, cells were treated with desipramine for 12 h, followed by an additional 3 h of TRAIL treatment for evaluating cell viability. For western blot analysis, TRAIL was given for 2 h. We observed reduced TRAIL sensitivity in the DR5-silenced cells with desipramine treatment, while TRAIL sensitivity increased in NC siRNA-transfected cells (Figs. **4A**-**C**). Western blot results displayed downregulated DR5 expression in the DR5-specific siRNA-transfected cells compared to the NC-siRNA-transfected cells (Figs. **4B**,**E**). DR5 silencing resulted in low expression of the apoptosis indicator proteins (cl-cas-8 and cl-cas-3), further supporting the significance of DR5 upregulation by desipramine in attenuating TRAIL resistance.

### 3.5 Effects of Desipramine Treatment on Autophagic Flux

**Fig. (5):**
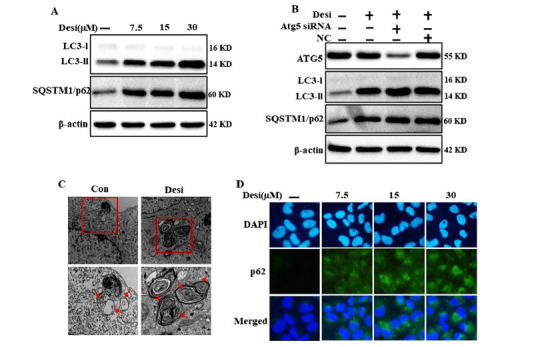
Effects of desipramine treatment on autophagic flux. A549 cells were incubated with designated doses of desipramine for 12 h. (**A**) Expression levels of LC3-II and p62 were analyzed by western blotting. (**B**) p62 expression on desipramine treatment was analyzed by ICC. (**C**) TEM showed an accumulation of autophagosomes. (**D**) The increased level of p62 expression by desipramine was evaluated by immunocytochemistry. Every experiment was repeated three times for (Fig. **A**) and twice for (Figs. **B**-**D**).

To identify the function of desipramine in autophagic flux, we targeted the autophagy markers LC3-II and p62 detection through western blotting. Immunoblotting assay revealed that desipramine inhibited lysosomal degradation of autophagy vesicles autophagic flux. We found that desipramine increased the LC3-I to LC3-II conversion, which indicates the formation of the autophagosome. Desipramine also increased the p62 levels as lysosomal degradation was inhibited (Fig. **5A**). The genetic autophagy inhibitor atg5 did not alter the p62 and LC3-II levels. Therefore, atg5-independent autophagosome accumulation occurred in desipramine-treated cells (Fig. **5B**). The transmission electron microscopy (TEM) study revealed the condensed accumulation of autophagic vacuoles. This was absent in the control, thereby establishing that desipramine inhibited autophagic flux (Fig. **5C**). ICC analysis also revealed that autophagic flux inhibition by desipramine was indicated by elevated p62 expression level dose-dependently (Fig. **5D**). These findings indicated that desipramine cause impairment of autophagic flux by disrupting the fusion of autophagosome and lysosome in lung cancer cells.

### 3.6 Autophagic Flux Inhibition Upregulated DR5

**Fig. (6):**
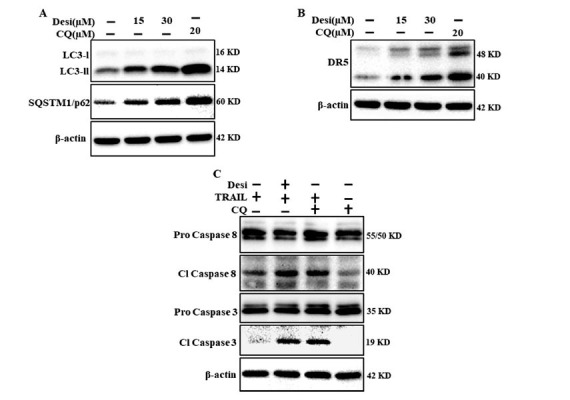
Autophagic flux inhibition upregulates DR5. Cells were preincubated with CQ (20 μM) and different doses of desipramine for 12 h. (**A**) LC3-II and p62 expression levels were evaluated by western blotting. (**B**) DR5 expression was assessed by immunoblotting. (**C**) Cells were preincubated with desipramine (30 μM) or CQ for 12 h and then treated with TRAIL (100 ng/mL) for 2 h. Western blotting technique was applied to evaluate the expression levels of cleaved caspase-8 and cleaved caspase-3. Every experiment was repeated twice.

To investigate the role of autophagic flux inhibition in DR5 expression, we used the autophagy inhibitor CQ. CQ inhibited autophagic flux and upregulated DR5 expression [58]. Cell culture plates were pretreated with CQ (20 μM) or different doses of desipramine for 12 h. Immunoblotting assay revealed elevated levels of LC3-II and p62 by desipramine and CQ, which suggests autophagy impairment (Fig. **6A**). Furthermore, both desipramine- and CQ-treated cells displayed induced DR5 expression compared to the control (Fig. **6B**). Finally, we discovered an increased caspase cleavage in both desipramine and CQ-treated cells in combination with TRAIL (Fig. **6C**). Overall, we proved that autophagy inhibition enhanced TRAIL-mediated apoptosis by upregulating DR5 expression.

### 3.7 Autophagy Inhibition by Desipramine Augmented TRAIL-Induced Cell Death

**Fig. (7):**
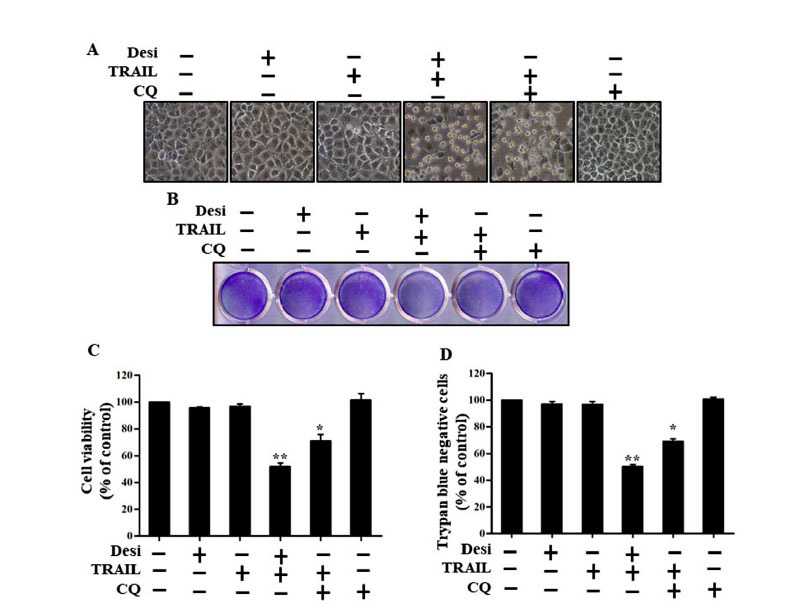
Autophagy inhibition by desipramine augments TRAIL-induced cell death. Cells were preincubated with or without CQ (20 μM) and desipramine (30 μM) for 12 h and finally exposed to TRAIL (100 ng/mL) for 3 h. (**A**) Cell morphology was captured under a light microscope (×100). (**B**) Mean density of crystal violet stained A549 cells is shown. (**C**) MTT assays were conducted to analyze cell viability, as represented by a bar diagram. (**D**) Viable cells were counted by trypan blue exclusion assay. Statistically significant differences between the control and each indicated treatment group are shown as *p <0.01 and **p <0.001. CQ: chloroquine. Every experiment was repeated three times.

We analyzed the role of desipramine in autophagy inhibition and subsequent TRAIL-mediated cell death by applying a functionally active autophagy inhibitor CQ. Cells were exposed to CQ or desipramine for 12 h and further incubated with TRAIL for 3 h. Through morphological analysis of cells using a light microscope and crystal violet assay, it was revealed that A549 cells treated with either TRAIL or desipramine showed mild cytotoxicity, whereas cells treated with a combination of desipramine or CQ and TRAIL showed significantly improved TRAIL-mediated cell death (Figs. **7A**, **B**). MTT and trypan blue staining assays demonstrated that cells subjected to combined treatment with desipramine or CQ (chloroquine) and TRAIL exhibited reduced cell viability and enhanced cell death (Figs. **7C**, **D**). Collectively, these findings showed that desipramine enhanced TRAIL-induced apoptosis by inhibiting autophagic flux at late-stage.

## 4 DISCUSSION

Depression is the most common symptom in cancer patients, and it suppresses their anti-cancer immunity [59-61]. Our primary objective of this study was to understand the role of desipramine and the co-treatment of desipramine and TRAIL in A549 lung cancer cells. We demonstrated that desipramine inhibited autophagic flux, resulting in DR5 upregulation. Consequently, this enhancement of DR5 expression ultimately augmented TRAIL-induced apoptosis in A549 cells.

TRAIL, a transmembrane cytokine, has shown potential in anti-cancer activities in tumor cells without cytotoxic effects [62, 63]. Due to its safety and potent biological properties, there is potential for the utilization of TRAIL as a viable agent in human cancer therapy [64, 65]. Despite that, the observed TRAIL resistance in certain cancer cells remains unclear. Autophagy serves a crucial role in recycling cellular components, where complete autophagic flux involves the recruitment of cellular components to lysosomes for degradation [66, 67]. Existing literature suggests that the activation of autophagy in cancer cells contributes to their resistance to TRAIL [68, 69]. Notable pharmaceutical agents, such as chloroquine (CQ) or hydroxychloroquine (HCQ), function as autophagy inhibitors [19] and have demonstrated the ability to impair autophagy in clinical trials aimed at cancer therapy [70, 71]. Recent studies suggested that autophagy inhibition sensitized cancer cells to apoptosis, and a complete autophagic flux promoted cancer cell survival [72, 73].

A549 lung cancer cells showed resistance to TRAIL treatment [19, 74]. Our present study established that desipramine or TRAIL alone could not induce cytotoxicity in the A549 cells. Significantly, the combination of desipramine and TRAIL had a remarkable effect on augmenting cell death in A549 cells. Moreover, the combined treatment robustly inhibited colony formation and reduced size in A549 cells (Figs. **1**, **2**). Desipramine, which upregulated DR5 expression, exerted this apoptotic effect owing to the combined effect of TRAIL and desipramine (Fig. **3**). This experiment proposed that desipramine, in combination with TRAIL, plays a role as an anti-cancer agent that can be employed to enhance the sensitivity of lung cancer cells towards TRAIL-induced apoptosis. Desipramine treatment at different doses in A549 cells increased LC3-II and p62 levels.

Our findings demonstrated that the inhibition of DR5 expression by DR5-specific siRNA abundantly increased cell viability and thus inhibited the effects of desipramine on TRAIL-mediated apoptosis. This suggests that the upregulation of DR5 is essential for the synergistic effect of desipramine and TRAIL combined. Moreover, these findings, for the first time, revealed that desipramine enhanced DR5 expression *via* autophagy inhibition (Fig. **4**). Upregulation of LC3-II indicates the accumulation of autophagic vacuole, and induced p62 level points to the disruption of autophagy at the late stage; that is, lysosomal degradation is interrupted [75]. We confirmed that exposure to desipramine induced autophagosome accumulation, which ultimately resulted in impaired autophagic flux (Fig. **5**). Additionally, the combination of treatment of desipramine or CQ with TRAIL increased cell death to a greater extent compared to individual treatments. Autophagy inhibition by both desipramine and the lysosomal inhibitor CQ upregulated DR5 expression level that effectively improved TRAIL-induced caspase-dependent apoptotic cell death. This was validated by the substantially increased levels of the intracellular apoptosis-related proteins, activated caspase-3 and activated caspase-8 (Figs. **6** and **7**).

## CONCLUSION

In conclusion, we reported that desipramine treatment enhanced the function of TRAIL by DR5 upregulation, which was facilitated by the inhibition of autophagic flux at the late stage. Desipramine and TRAIL in combination stimulated apoptosis in TRAIL-resistant A549 cells, suggesting that desipramine treatment enhanced TRAIL-induced cancer cell death, specifically in TRAIL-resistant lung cancer cells. The findings deserve advanced studies on cancer patients to confirm the role of autophagic flux in DR5 in relation to TRAIL-mediated cancer therapy. However, this study could be the basis for future studies in choosing treatment options for patients suffering from both cancer and depression.

## Data Availability

All datasets generated or analyzed during the present study are available from the corresponding author upon reasonable request.

## LIST OF ABBREVIATIONS

CQ: Chloroquine
DISC: Death-inducing Signaling Complex
DR /5: Death Receptor 4/5
FADD: Fas-associated Death Domain
ICC: Immunocytochemistry
LC3: Microtubule-associated Protein Light Chain 3
MTT: Methyl Thiazolyltetrazolium
siRNA: Small Interfering RNA
TCA: Tricyclic Antidepressant
TEM: Transmission Electron Microscopy
TRAIL: Tumor Necrosis Factor-related Apoptosis-inducing Ligand
